# Between War and Peace, Past and Future: Experiencing the Hiroshima Peace Memorial Park

**DOI:** 10.1007/s12124-022-09723-2

**Published:** 2022-10-20

**Authors:** Ignacio Brescó de Luna, Yuanhang Li, Brady Wagoner

**Affiliations:** 1 Universidad Autónoma de Madrid, Madrid, Spain; 2 Kyushu University, Fukuoka, Japan; 3 Aalborg University, Aalborg, Denmark

**Keywords:** Hiroshima Peace Memorial Park, Memorials, Subjective camera, A-Bomb, Memory politics, Themata

## Abstract

Hiroshima Peace Memorial Park is widely known as a universal symbol of peace, but there have not been studies of how people actually experience and interpret it. This article presents a detailed case study of a visit to the memorial by using an innovative methodology based on the use of subjective cameras (subcams). Results show that despite the monolithic idea of peace that the memorial officially represents, it is experienced and interpreted in terms of a constant tension which exposes conflicts in post-war Japan memory politics. The dichotomies of war/peace, death/life, past/future, and old /new emerge as part of the participant’s encounter with different situations during his visit. This is particularly clear where he perceives border zones and points of intersection. The article concludes by interpreting these dichotomies through the notion of themata, as elementary dichotomies that underlie a social debate around a specific topic. Specifically, two themata are proposed: one revolving around the temporal problematisation of the past and the future in the memory politics of the A-Bomb, and the other revolving around the spatial dichotomy between the old and the new underlying Hiroshima’s urban renewal.

On 6 August 1945, an American B-29 bomber dropped an atomic bomb on the city Hiroshima. The explosion razed the city to the ground and caused the deaths of around 140,000, as a result of direct and indirect effects.^1^ On the fourth anniversary of the bombing, the architect Tange Kenzo (1913–2005) was appointed to carry out his plan for the Hiroshima Peace Memorial Parkl (HPMP) in the Nakajima district, close to the explosion’s hypocentre. Dedicated to the memories of the victims and survivors of the bomb, this park –home to iconic landmarks, such as the ruins of the A-bomb Dome or the Hiroshima Peace Memorial Museum– has become a universal symbol of peace for which the city is known today, drawing over a million visitors each year. Despite this, when Tange asked himself “what crosses people’s minds when they stand in the park?” he answered that “it might vary from individual to individual” (Maki & Niihata, 2020, p. 9). 75 years later Tange’s question lives on, especially as fears of a new nuclear escalation loom once again.

This article presents a detailed case study of experiencing and interpreting the Hiroshima memorial. Based on a previous line of research (Brescó & Wagoner, 2019a, b; in press; Brescó et al., 2020), our fieldwork at HPMP sets out to elaborate Tange’s answer by asking: How does the HPMP’s design feed into the way in which people experience the site? What elements stand out and why? What meaning-making processes do these elements elicit during the visit? These are relevant questions after 75 years marked by an overly idealistic symbolism of peace that has rendered a monolithic character for the city of Hiroshima, thus silencing a number of controversies and contradictions about the way the a-bomb has been officially remembered (Minami & Davis, 2018).

Whereas previous research on memorials has mainly focused either on architectural features or observations of people on-site (e.g., Stevens & Franck 2015), our focus is on the situated, ongoing interaction between individuals and memorial sites from the participant’s own perspective. In line with new methodological approaches, such as the sensory and video ethnography (Pink et al., 2017), we are interested in studying the visitors’ contextualized meanings and feelings at these sites, including their atmospheric qualities (Sumartojo & Pink, 2019). To this end, we propose an innovative methodology based on the use of subjective cameras (subcams), particularly tailored to study the possibilities offered by different memorials (see Wagoner et al., 2022). In what follows, we first outline some key features of Hiroshima Peace Memorial Park , controversies surrounding it and our theoretical framework for understanding visitor’s meaning making. Second, we provide an in-depth case study of a person’s trajectory of experience through the memorial. This analysis will focus on visual perception of borders and intersections on the one hand, and conceptual distinctions on the other.

## Politics of the Hiroshima Peace Memorial Park: A Brief History of Ambivalence

Memorial sites involve a process of symbol formation aimed at commemorating collective events, including loss and trauma (Minami & Davis, 2018). Conceived as spaces of shared memory, memorials provide a physical site to express and emotionally connect with the collective loss, which helps individuals and societies to reinterpret the past, and in so doing construct new orientations to the future (Wagoner, 2017). As cultural artefacts, memorials have undergone multiple changes throughout history. Traumatic events in the 20th century –such as the two World Wars, Auschwitz, or Hiroshima and Nagasaki– disrupted the functionality of traditional memorials, typically characterised by a vertical and an affirmative style that features conventional symbols and figurative representations of heroes and martyrs. The forms in which collective loss had been so far represented and socially remembered were called into question and became insufficient to memorialize events felt to be unrepresentable and unthinkable (Young, 2000). In the absence of a narrative capable of conferring a clear and specific meaning to the past, memorials in the second half of the last century increasingly turned to an abstract, non-representational and non-figurative style. This ‘counter’ memorial form tends to invite people to actively search for their own meaning to the site (see Brescó & Wagoner 2019a).

Bull & Hansen (2016) associate the abstractness of counter memorials –such as the famous Maya Lin’s Vietnam Veterans Memorial in Washington D.C.– with what they call the *cosmopolitan mode of remembering*, as opposed to the *antagonistic mode of remembering* that is typical of traditional memorials. Whereas the latter tends to represent the past in terms of moral categories – ‘good’ vs. ‘evil’ – applied to specific groups, the abstract style of the former, “with its emphasis on the unknowability the unspeakability of traumatic events” (Bull & Hansen, 2016, p. 395), aims to transcend historical particularism with its focus on human suffering. As such, moral categories no longer refer to concrete groups but to universal values, such as peace vs. war. However, according to these authors, the victim-centred approach of counter memorials tends to hide the memory politics of the commemorated event under a seeming social consensus.

In the case of HPMP, the promotion of peace as universal value, and as a response to the global threat to human civilization posed by nuclear weapons, is meant to transcend political and geographical frontiers. This position, referred to as *nuclear universalism* by Yoneyama (1999), implies, according to this author, remembering Hiroshima’s bombing “from the transcendent and anonymous position of humanity” (p. 11). This mode of remembering is eloquently illustrated by the controversy over the epitaph etched on the Cenotaph situated at the heart of the HPMP. Covered by an arch-shaped monument representing a shelter for the victims’ souls, the official English translation reads, “Let all the souls here rest in peace, for we shall not repeat the evil”. However, in the original Japanese, the second sentence lacks a grammatical subject, thus leaving responsibility for the evil to a subject-less humanity (see Minami & Davis 2018).

This controversy around the epitaph shows how, although the original purpose behind the HPMP was to create a consensual urban space around the notion of peace, “it eventually became a site for conflict, an ambivalent place” (Schäfer, 2008, p. 168). As Yoneyama (1999) points out, this ambivalence can be found behind the tendency to conflate two seemingly opposing signs, “A-bomb” and “Peace”—a tendency apparently encouraged by the Allied, then occupying forces in Japan. Thus, “the park is named Peace Memorial Park, rather than Atom Bomb Memorial Park” (p. 161). According to this author, the notion of peace in this context mainly refers to post-war recovery, a period associated with a bright future of progress or, as Schäfer (2008) puts it, a period “defined in accordance to what it was not” (p. 155), that is, in opposition with a past of war and destruction. In this sense, in Schäfer’s words (2008), “probably no war memory reflects the dualism of present and past, peace and war so fiercely as the memories of the first atomic bombing – Hiroshima, 6 August 1945” (p. 155). Another example of this dualism and ambivalent tension can be found in Hiroshima’s post-war image of peace-loving and victimized city vis-à-vis its prior role as a flourishing military centre in Imperial Japan, and more particularly, in its contribution to the war effort during the colonial expansion in Asia and World War II.

The tension between contrasting opposites that cuts through the A-bomb’s memory politics materially translates into the spatial politics of Hiroshima’s urban renewal. According to Yoneyama (1999), this dualism is mainly present in the way “different urban topographies […] are defined by dissonant temporalities” (p. 34), whereby the city’s dark past of war and death associated to the HPMP stands in stark contrast with a bright, cheerful and weightlessness celebration of progress. As the author goes on to say, “a large part of the production of Hiroshima’s “bright” new memory-scape involved the clearing away of physical reminders of the war and atomic destruction” (p. 48). This generated a debate over the fate of the ruins and those architectural remains that withstood the A-bomb, a debate on whether to demolish them in the interest of the city economic recovery or to preserve them without any utilitarian function as relics of a painful past. Unlike buildings such as the Nippon Bank Building or the Red Cross Hospital, rehabilitated after the war, the iconic Atom Bomb Dome (*Genbaku Dome*) became a musealised object, which stands today as a material example of the duality that looms behind the memories of the a-bomb in Hiroshima. Visible from the memorial’s Cenotaph and with a central role in Tange’s design for the HPMP, the skeletal remains of the former Industry Promotion Hall stand in stark contrast to the modern city background. However, in addition to bearing a fragment of a past fraught with death and destruction –which contrasts with Hiroshima’s post-war rebirth– the ruins of the A-Bomb Dome also bear witness to the city’s pre-war times by showing a fragment of “a quintessential sign of Japan’s early-twentieth-century imperial modernity” (Yoneyama, 1999 p. 2).

Tensions between striving for a future recovery and the unbearable weight of the past are always present in debates around memorials or those remains left after collective traumas (see Andriani & Manning 2010), just as memory politics are inevitably tied to the dialectics of remembering and forgetting (Assmann, 2012; Brescó, 2019). In the case of the A-bomb memories, the apparent consensus behind the HPMP as a universal symbol of peace seems to obscure certain underlying tension in what Yoneyama (1999) deems an effort at taming the city’s memory-scape or repressing some of its painful memories (Minami & Davis, 2018). These tensions looming behind the representation of A-bomb memories in HPMP –tensions between past and future, war and peace, death and life, creation and destruction, perpetrators and victims, memory and forgetting– can be understood through the notion of *themata*. Themata are mutually interdependent antinomies that have been thematised through history (Markova, 2000; Moscovici & Vignaux, 2000). For instance, the oppositional pair of yin/yang is used to account for opposing and interdependent forces in Chinese cosmology. The concept of themata originally comes from the philosophy of science, where Holton (1996) used it to look at the basic distinctions, such as continuity/discontinuity, out of which scientific theories are constructed –for example, contemporary theories of atoms draw on the same themata that the pre-Socratic philosopher Democritus used in Ancient Greece. From the framework of social representations theory, themata help to understand the dialogical dynamics of common sense thinking and its embeddedness in history (Moscovici & Vignaux, 2000).

Thus, due to a crisis or an unexpected event, some implicit dichotomies in our common sense become themata by being problematised and exposed to social attention and public debate (Markova, 2000). For instance, the notions of justice/injustice are dialogically discussed and reconstructed in the context of the Israeli-Palestinian conflict (Nicholson, 2019), just as concepts of life/death are within organ donation and transplantation debates (Moloney et al., 2011) to mention but a few examples. This approach can be applied to understand the tensions that characterise the way in which the A-bomb has been socially remembered, debated, and represented in post-war Hiroshima. Following Moscovici (1993), we could say that themata act as conceptual coat hangers that provide socially generated ways of understanding the A-bomb and its memory. In this way, individuals may go from implicitly using these antinomies –thus embracing themata in their discourse without being fully aware (Rochira, 2014)– to problematise and reflect on them, thereby expressing their “effort to understand and appropriate meaning” (Markova, 2000, p. 454). As we will show in the study that follows, experiencing HPMP will give rise to an interpretation of the site in terms of a constant tension between opposites, thus exposing some of the tensions behind the monolithic idea of peace this memorial officially represents.

## Methodological Approach

Studying how people experience memorials requires going from traditional mono-modal approaches –based on verbal data detached from contextualised activity– to processual approaches capable of capturing individuals’ multi-modal forms of experience and meaning-making, as part of a wider set of movements and interactions in space. One of the most recent additions to this area has been the use of subjective cameras (subcams), which record individuals’ ongoing experience from a first-person perspective, in both video and audio (Lahlou, 2011). Subcams, in combination with interviews, offer one of the most contextualised, socio-material, holistic, multisensory, and process-focused data collection devices currently available. In the particular context of the fieldwork at HPMP, the use of the subjective camera was combined with a post-visit interview in which the subcam recorded footage was utilized as a video-elicitation tool in line with other video ethnographic approaches (Pink et al., 2017).

The fieldwork was conducted on 3 December 2021 on the occasion of a research stay by the first author at Kyushu University, hosted by Prof. Minami Hirofumi and funded by the Japan Society for the Promotion of Science. 2 Drawing on previous works by the first and the third authors, the study was planned in collaboration with Prof. Minami, with the second author –student of the master’s degree in Kansei Science at the School of Integrated Frontier Sciences, Kyushu University– assuming the role of single participant and interviewee and the first author the role of interviewer. At the time of the study, the second author was completing his master’s thesis under Prof. Minami’s supervision on the topic of Genius Loci – i.e., the spirit of a place according to the ancient Roman tradition. Due to the common interest of the first and second authors in studying the atmosphere of places, Prof. Minami suggested the latter as a possible participant in the study.^3^ The only instructions given to the second author (henceforth SA) were to walk freely through the memorial alone with the subcam, thus giving total autonomy to experience the place. For instance, he was also free to interact with other visitors, which the subcam would have audio registered, although he did not talk to anyone during his visit. After the visit, the resulting video recordings were replayed back to the SA in a post-visit interview conducted by the first author (FA) at an off-site location.

While watching the subcam video of their visit, the SA was able to comment on the experience by reflecting on his affective engagement with the environment and the meanings and associations afforded by some of its elements. As became evident in our previous fieldwork conducted at the Memorial of the Murdered Jews of Europe in Berlin (Brescó et al., 2020), the post-visit interview is a necessary complement to subcam data because not everything in a person’s visual field is registered or actively attended to as a meaningful component of their experience (Gibson, 1986; von Uexkull 1992). Furthermore, in human perception what becomes a focal point of experience is often symbolically elaborated in reference to the cultural world to which the person belongs as well as their personal history (Valsiner, 2014). This signals a shift from the direct to indirect (or symbolic) perception of the environment (Heft, 2001), expressed in the participant’s associations and reflections about the site during the interview.

The post-walk interview was transcribed and thematically coded by the FA. The themes associated with different strings of antinomies regarding the HPMP quickly emerged as key in the SA’s experience of the memorial. For each quotation regarding each antinomy, we identified the corresponding moment in the subcam video and took a screenshot of it. Screenshots from the subcam video will be shown to illustrate features of memorials from the SA’s standpoint, and thereby highlight contextual and experiential qualities, powerfully captured through visual methods.

Both when planning the research and after the data collection at HPMP, specific ethical challenges posed by wearable cameras (Mok et al., 2015) were discussed and addressed, particularly those involving minimising the scale and scope of data featuring third parties – and promoting participant’s control over visual data, including access to or withdrawal of the data as part of the ongoing process of consent. Furthermore, in line with what Sumartojo & Pink (2019) highlight in their studies, the fact that the FA also visited the memorial, provided him with a basis upon which to empathetically discuss the experience with the SA.

## A Case Study of Visitor Experience in Four Phases

**Fig. 1:**
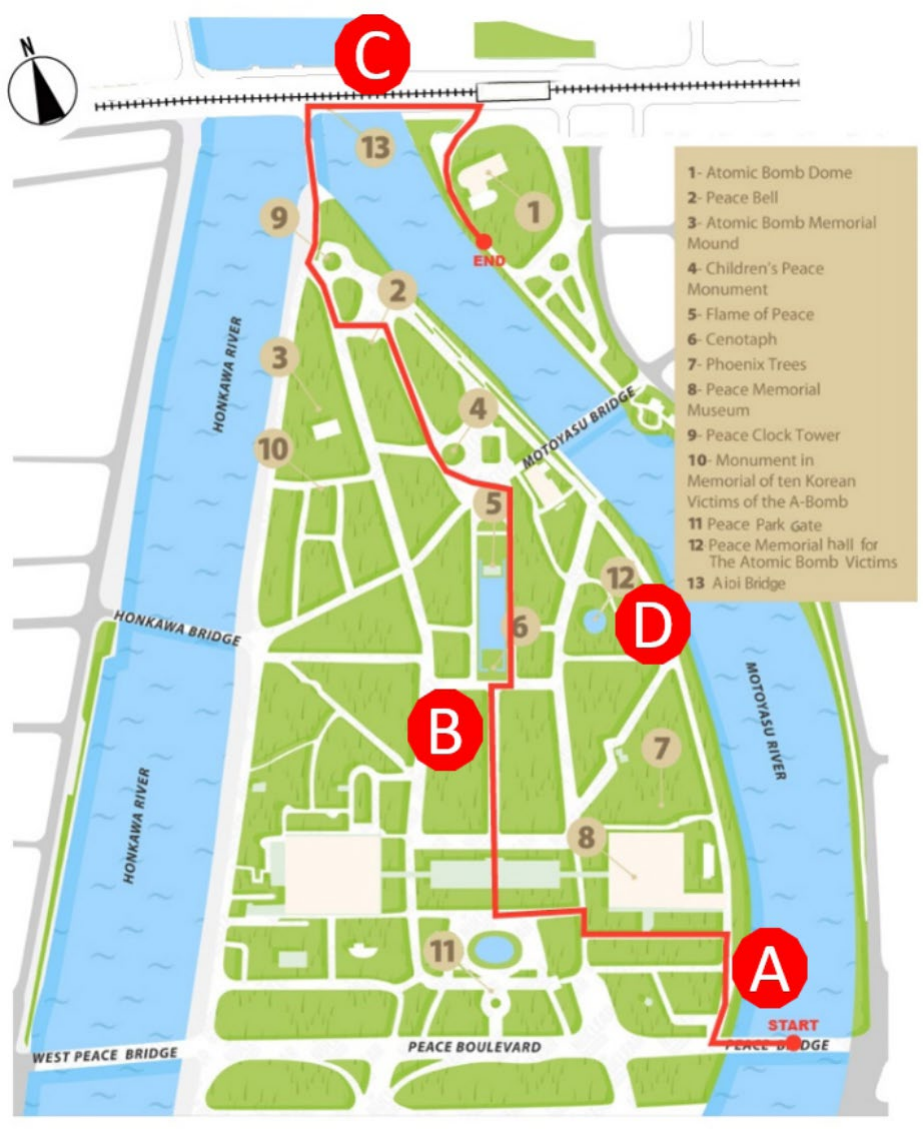
Hiroshima Peace Memorial Park map, including the participant’s trajectory into the site divided into four phases (A, B, C, D)

On December 3, 2021, the FA and the SA –accompanied by prof. Minami Hirofumi from Kyushu University– took a Shinkansen from Hakata train station in Fukuoka bound to Hiroshima. After checking in at a hotel nearby, we took the Peace Boulevard to start the visit to the HPMP from the southern side. This decision was advised by Professor Minami, as both the FA and SA had already visited the memorial some years ago starting from the north side, where the A-bomb dome is located. Once we crossed the bridge over the Motoyasu river, the SA began his visit alone equipped with the subcam glasses. The analysis that follows focuses on three moments of the visit around which the subsequent post-visit interview revolved: (a) the memorial entrance, (b) the Cenotaph and (c) the A-Dome. In subsection (d) we include the analysis of the SA’s visit to the Hiroshima National Peace Memorial Hall for the Atomic Bomb Victims, which took place the following day without the subcam glasses. Figure 1 features HPMP’s map, including SA’s trajectory into the site divided into these four phases.

*1) Entering the memorial: a transition from the mundane to the sacred*.

When the FA and the SA parted ways upon crossing the bridge, the latter initiated the visit by deciding not to go straight to the memorial’s main entrance –where the Gate of the Peace is. He took, instead, the path along the riverside bordering the memorial (see point “A” in Fig. 1). While watching the subcam video featuring the tree-lined path along the river, the SA explains his decision of delaying the encounter of the memorial’s central area:

“Coming to the park from this way, mmm … it is quiet at first. So… your emotions have time to attune to the site, instead of going directly to the memorial’s centre. In this way you are more in control… you do the visit at your own pace”.

He then goes on to compare entering the memorial with entering a sacred space for which one needs to go through a series of rituals to purify oneself in order to be prepared both mentally and emotionally: “When you go to a Japanese shrine you have to wash your hands […] It is like going from everyday life world to a religious or sacred area”. In her *Purity and Danger*, Douglas (1966) argues that purification rites signal a border. Uncleanliness is a relative notion: Shoes on a clean carpet are dirty but not outside. The ritual of taking of shoes to enter the house is indicative of moving through a border.

As we can observe at this initial stage of the visit, the location of the memorial at the crossroads of two rivers,^4^ creates a kind of border zone separating the city’s everyday life from a different area encapsulated within the HPMP. As a result, the path along the river is perceived as an intersection point between these two spaces, as a liminal zone in which to perform certain rites of passage to transit between two worlds charged with different spiritual and affective atmospheres. The perception of this border zone –which points to the city’s different “urban topographies” as noted by Yoneyama (1999)– marks an inside and an outside, thereby prompting the SA to interpret his experience through the antinomy *sacred*/*mundane*, which confers a value distinction between the inside and the outside (Tateo & Marsico, 2021).

**Fig. 2:**
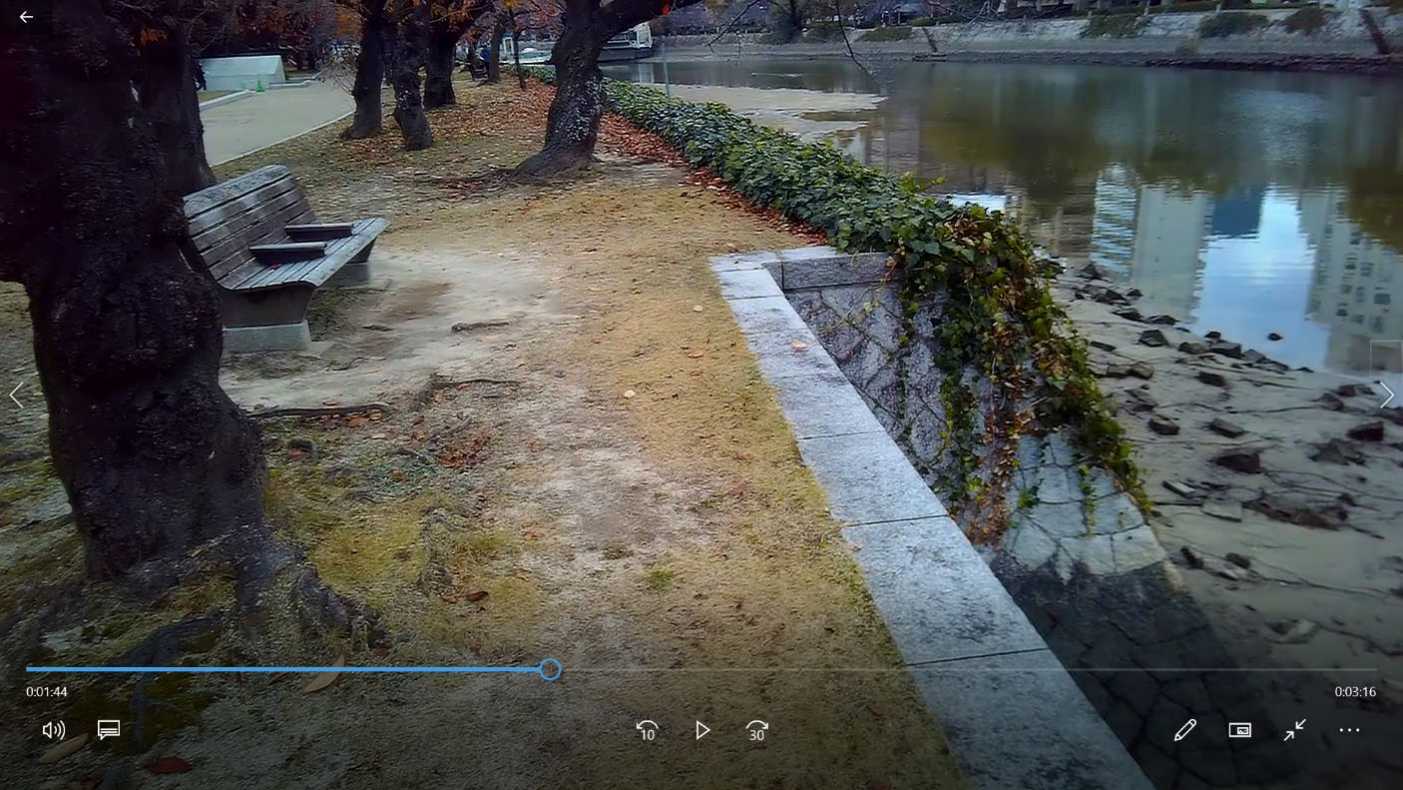
Screenshot from subcam video when the SA was referring to the natural life around the river

However, this value distinction between the inside of the HPMP’s area and the outside is reversed with the use of another pair of opposites which comes up immediately after in the interview. These antinomies emerge when the SA explains why he did not want to turn his gaze to the ruins of the A-Dome building –whose presence can be sensed in the background of the image– while walking along the river (see Fig. 2). In the words that follow, we can see a clear opposition between *war*, as a man-made *artificial* thing –represented by the image of the A-bomb Dome building– and *nature* –represented by the life around the river–, associated with normality, and by implication, with *peace*.

“Even though I could see straight at the dome… [but] I think I did not want the dome to get to my sight too quick, so I looked at something else […] I liked to concentrate on animals and nature, for example, the birds, the river… […] I think the war is made by humans. The war is not a product of nature, it is just a game of people. So, if you want to return to the normal, you should come back to nature”.

A few minutes later, the video shows the SA stopping on the way and turning in the direction of the central area of the memorial. In watching these images, he comments: “Here I thought it was time to put the real thing in front of me”.

*2) ‘Like a time machine’: between past and future, death and life.*

**Fig. 3:**
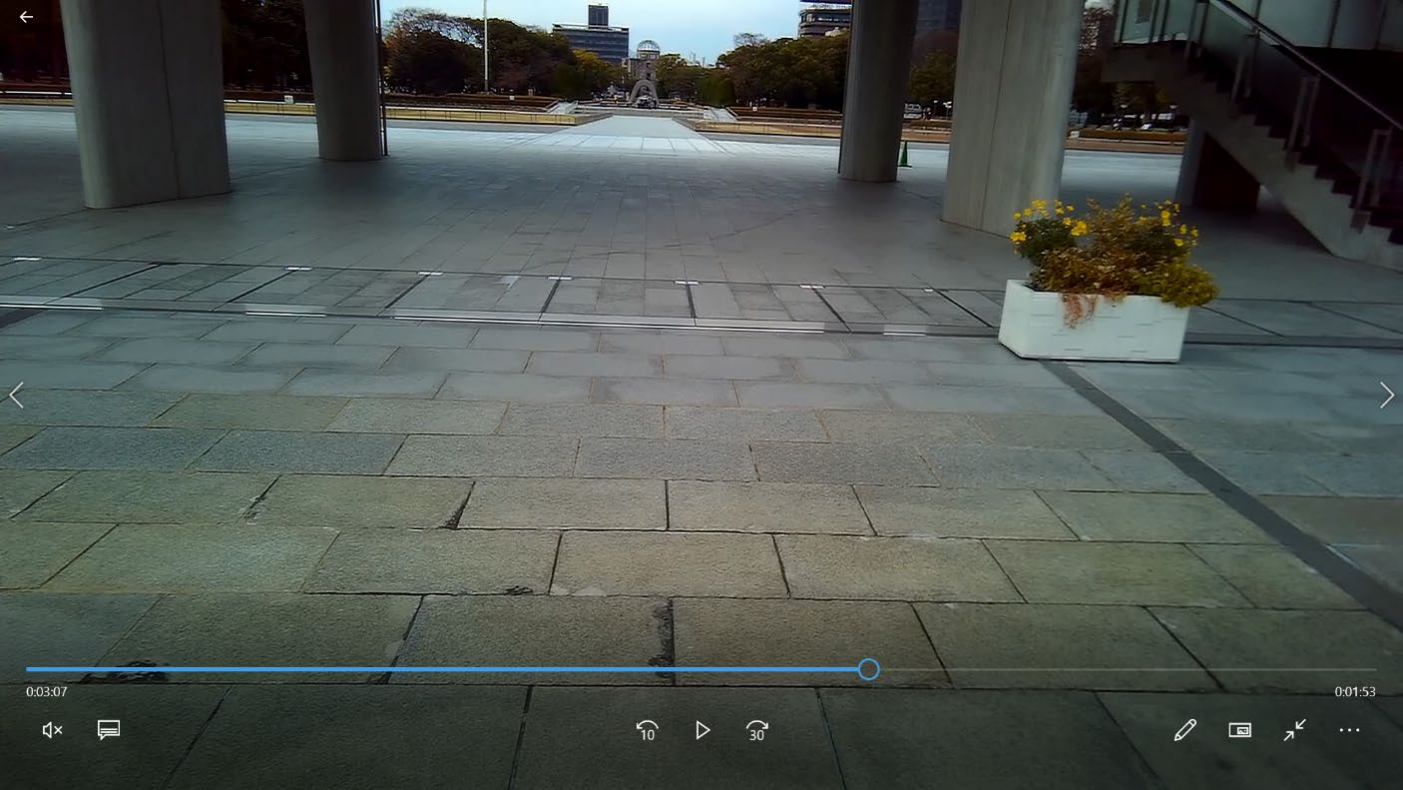
Screenshot from subcam video when the SA turned his gaze to the Cenotaph and the A-Dome in the background

While watching the video of himself heading towards the area where the Peace Memorial Museum is located, the SA comments on the sense of anticipation he was feeling at the upcoming appearance of the Cenotaph in the central part of the HPMP. This sense of expectation points to the emergent and flowing nature of atmospheres as a sensory quality of experience. As Sumartojo & Pink (2019) point out, the emergence of atmospheres “entails both a mode of experiencing the present moment, and anticipatory mode relating to what might come next and the feelings that this might involve” (p. 24). In the SA’s own words: “I know that I will see the monument and the A-dome very soon, so I am very excited”. Some seconds later, he describes his first reaction upon seeing the Cenotaph and the ruins of the A-dome looming in the background of the image (see Fig. 3): “When I turn right, at this moment I see the monument [the Cenotaph] and the dome […]. This makes me very emotional. It touches me a lot”.

The video shows how he stands contemplating the Cenotaph with the A-Dome in the background from below the Peace Memorial Museum for almost two minutes (see point “B” in Fig. 1). While watching this perspective on the screen, the SA highlights a sense of convergence between the trees and the Cenotaph pointing towards the A-Dome. He says: “This moment is the most touchable one, when I see the combination of the monument and the dome”. As we will see, this convergence will make the Cenotaph to be perceived as an intersection point between different opposing meanings.

This connection with the site is further reinforced by the happy atmosphere he experiences in the site due to the presence of people enjoying the park and children offering flowers at the Cenotaph, something the SA associates with life, hope and future. In his own words:

“The contrast between the history of 70 years ago and the presence of the children at this moment is striking […] You see a lot of children here offering flowers to pray for that people [who died]. I feel it is a nice moment because history and children make a very interesting combination. Children are a symbol of life, and, on the other side, you have the dead people. […] I also see a lot of people walking their dog or just sitting and talking with friends even though this is a site with a very sad history. But still, people use this space to chat. This creates a happy atmosphere”.

**Fig. 4:**
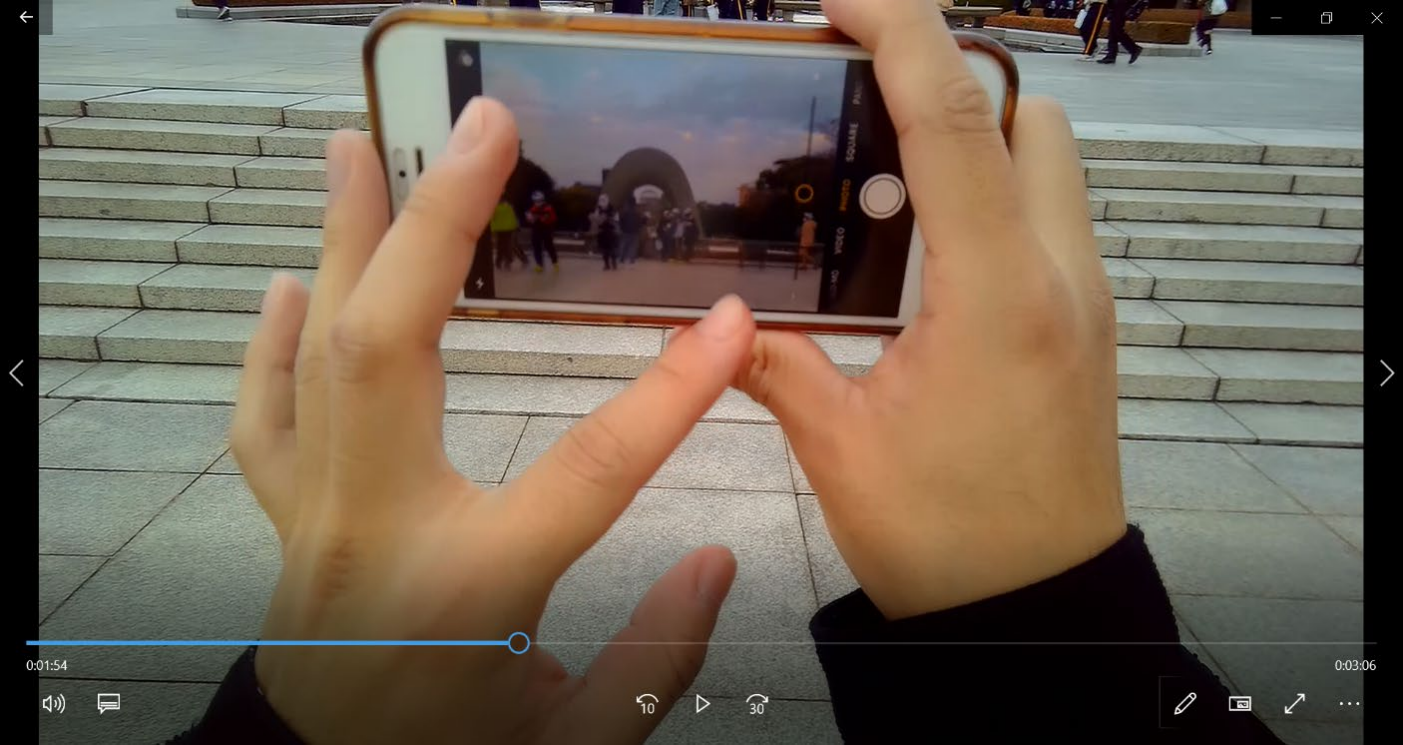
Screenshot from subcam video when the SA was taking a picture with the children by the Cenotaph

This fragment shows how people co-create atmospheres through their actions (Sumartojo, 2016). In this case, we can see how the atmosphere co-created by children offering flowers at the Cenotaph leads the SA to interpret the site through a string of connected antinomies opposing *life* –symbolically represented by children– to the *dead* people remembered through the monument, and the place’s *sad past* to the *happy* atmosphere the SA is experiencing in *present*. Interestingly, there is a moment in the subcam video where the SA appears taking a picture of the children around the Cenotaph (Fig. 4) in an attempt at capturing and making sense of that perceived dichotomy between the memorial, representing the dead and the past, and the children, representing life and future. In SA’s words: “I wanted to put the children and the monument in the same photo, combining these two elements [children and Cenotaph]”.

Capturing these antinomies in the picture becomes a meaning-making resource as it enables the scaffolding of different reflections anchored in the SA situated experience (see Jiménez-Alonso & Brescó, 2022). Echoing the social debate around the HPMP alluded to in a previous section, the SA argues: “I don’t think the monument should just tell people the sad things, but I think that… the monument should give people the hope for the future”.

However, this tension between the hope for future and the sadness of the past is reframed and given a personal meaning by turning it into an imaginary intergenerational dialogue. This interpretation seems to be elicited by the very disposition of the arch-shaped Cenotaph framing the A-dome in the background, thereby acting as symbolic and special intersection point between the past and the present, between the victims and the participant. As we can see in the following excerpt:

I felt that... if you stand in front of the monument [points to the Cenotaph] and the A-dome, is like you are talking to your grandfather or your grandmother, listening to stories from them. It is like being with your family

This imaginary dialogue was further supported by some sensory elements of the memorial, such as the fire of the Peace Flame. Symbolising the sea of fire that the city became after the bomb, the constant movement and regeneration of the flames leads this element to be perceived as one embracing both life and death, somehow signalling the present absence of the A-bomb victims. In watching the subcam video showing the Peace Flame framed by the Cenotaph, the SA comments:

Especially when I see the fire, which is constantly moving… I felt like something was alive. So, I imagined there were thousands of people just right there telling something to me, as if they were my grandpa or my grandma […] I got very moved by it

**Fig. 5:**
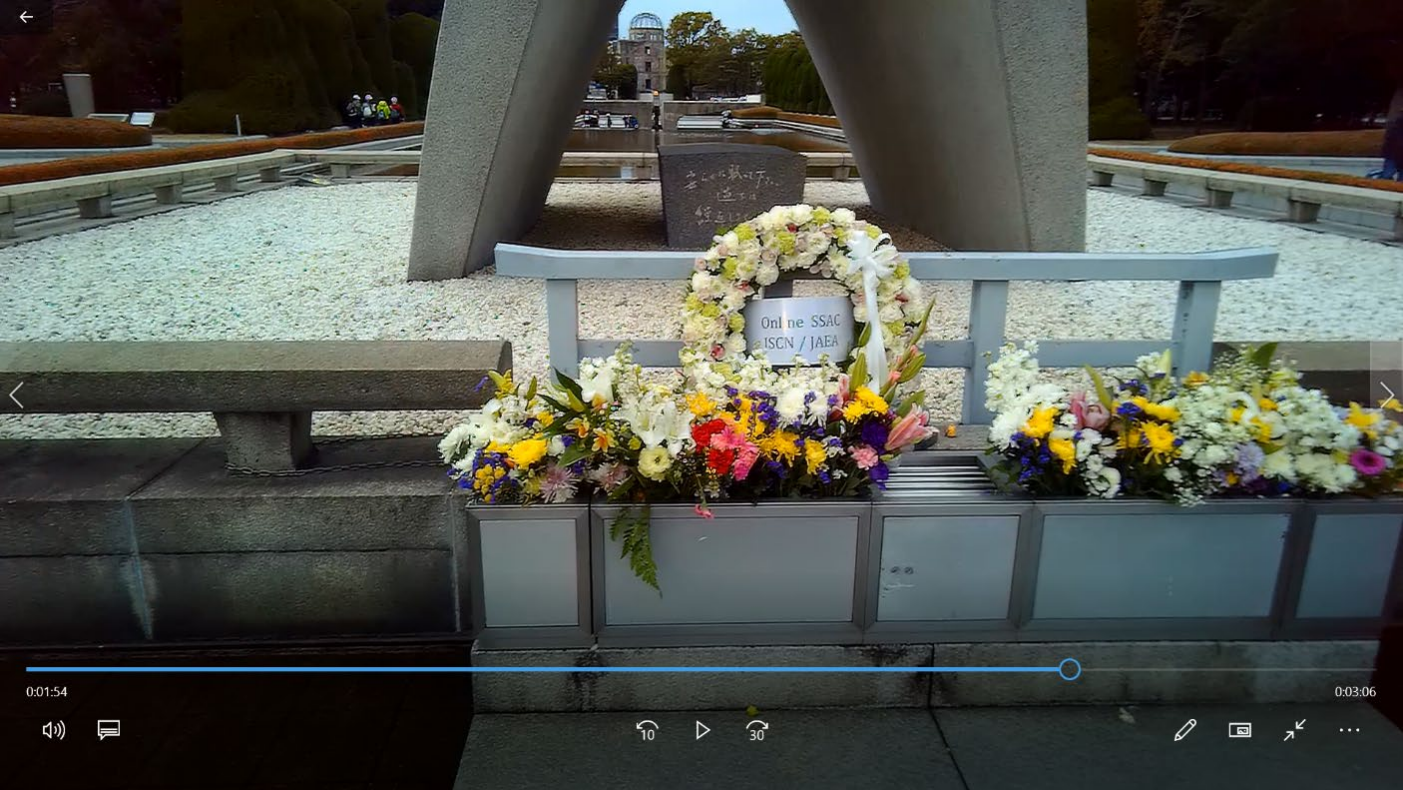
Screenshot from subcam video when the SA was referring to convergence between the Cenotaph, the Peace Flame (barely noticeable in the image), and the A-Dome

Finally, while watching the video featuring the A-Dome at the background framed by the Cenotaph, the SA highlights once more the convergence between the Cenotaph and the A-Dome forming a powerful symbolic axis (Fig. 5). It is precisely in reexperiencing this axial view through the video that the SA resorts to a metaphorisation of the place (Dekel, 2009). In the next excerpt we can see how the analogy between the place and a time machine summarises, to some extent, the SA’s experience of the memorial as a site that affords transiting between opposites, between the *present* and the *past*, between *life* and *death*. Here, it important to highlight the situated emergence of this metaphor triggered by the memorial layout and the movements of the SA in the memorial space. The very position and perspective from which the SA is looking at the A-Dome through the arch-shaped form of the Cenotaph seems to create an intersection point affording a visual connection and a connection of ideas.

The monument [the Cenotaph] is like an arch, so you can see through this monument and look at the dome. From this perspective, I can feel a gap in time; that side [pointing to the background of the image where the A-dome rears its head] is the past and this side [pointing the image featuring the Cenotaph in foreground] is the present. So, it is like a time machine that pushes you […] It is like a magnet that attracts you. You can feel this time gap in the design of the space

*3) Between the old and the new: the symbolic and material power of the A-Dome*.

**Fig. 6:**
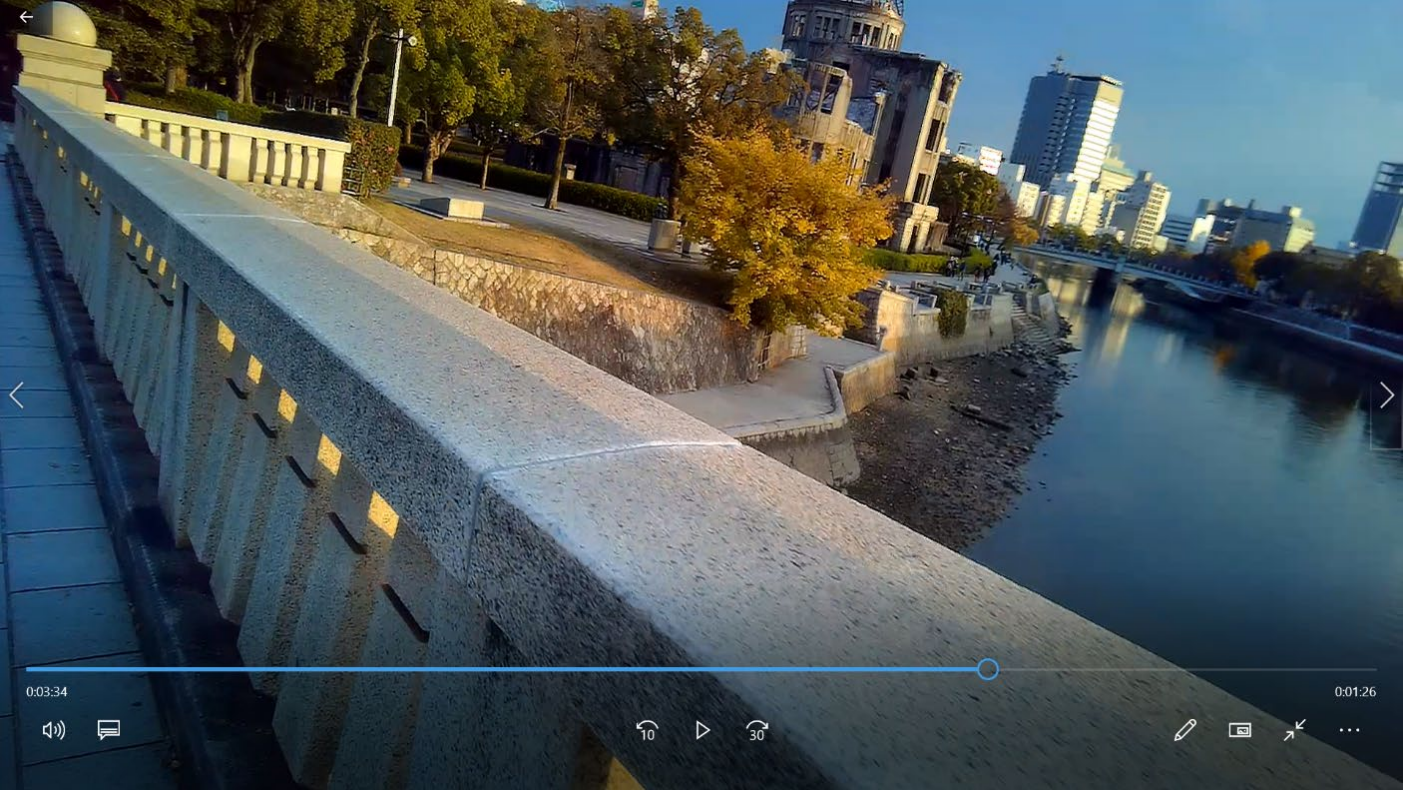
Screenshot from subcam video when the SA was referring to presence of the A-Dome seen from the Aioi Bridge

The interview goes on as the subcam video progresses showing images of the SA walking along the Peace Pond in the direction of the Children’s Peace Monument without paying much attention to it. After leaving behind that monument, he makes his way towards the twisted Peace Clock Tower which marks the exact time when the bomb was dropped on August 6, thereby bringing the tension between the present and past –made present through the hands of the clock– to the fore once again. From there, the video shows the SA crossing the Aioi Bridge^5^ (see point “C” in Fig. 1) with the A-Dome making its appearance on screen (Fig. 6).

Watching the sunset light casting on the A-Dome, the SA refers to the sacred atmosphere conveyed by these ruins, thus picking up again the duality between the sacred meaning of the HPMP and the mundane life outside the memorial. To this distinction the SA adds the stark contrast generated by “the building in ruins surrounded by more modern architecture”. This is a contrast –between the old and the new, between the city’s past and its modern present– that, in the words of the SA, makes the A-Dome to “stand out more from its surroundings”. Furthermore, the objectification of the past through the A-Dome’s ruins creates a particular atmosphere that feeds into the SA’s imagination when he begins to talk about the people working in the former Industry Promotion Hall: “The people in the building back in 1945 maybe they were busy going about their business and had no time to scape and then vanished in one second”. As Beckstead (2017) points out, the past evoked by the ruins affords individuals “to be affectively drawn into the setting in the here-and-now and to go beyond it and to imagine what life was like for those who lived, worked, loved in, around, or near the build that has become a ruin” (p. 133).

**Fig. 7:**
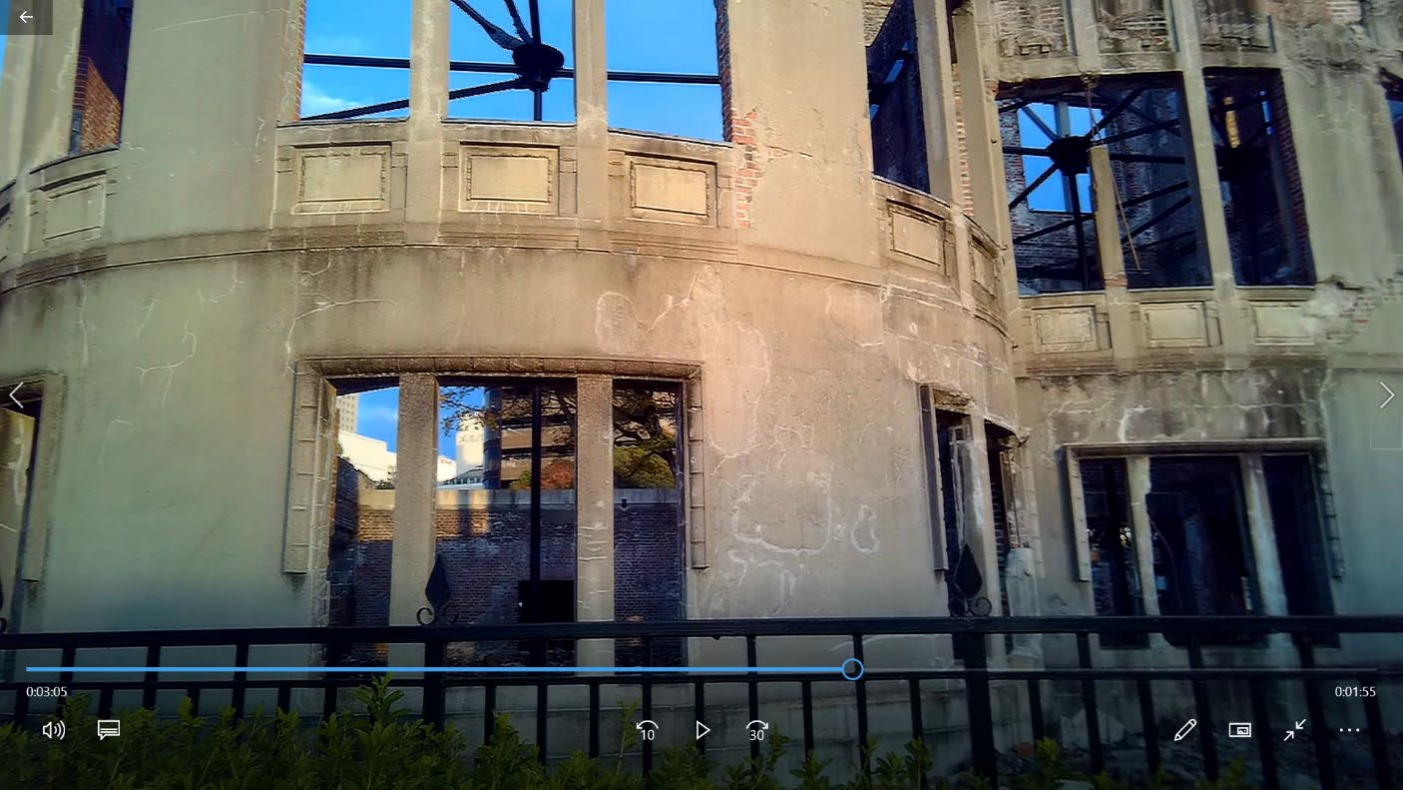
Screenshot from subcam video when the SA began to engage in a more detailed description of the A-Dome’s material aspects

Drawing on Niels Bohr’s observations during his visit to Hamlet’s castle in Kronborg, Denmark, Beckstead (2017) stresses the fact that ruins are more than a material object composed of stones, but are places saturated with meaning. This assertion is behind the way in which the SA reflects on the meaning of the A-Dome while watching this building through the subcam video. At first, he remarks that “the building is not as strong as when seen from the Cenotaph perspective. When seen from that perspective you see a monument, but here I… just see a building in its ruins”. However, as he watches the recorded images of the ruins in more detail (Fig. 7), he starts paying attention to the building’s specific features:

You can see how the windows are all blown out and the iron has been twisted, so you can feel the strong wind that came after the bomb […] It’s like a sculpture shaped by the wind of the A-bomb […] This makes this place to have a very special atmosphere

And from that point, he begins to reflect on the complexity of the building’s entrails:

Looking at the A-Dome from far away it becomes a symbol, but if you come closer, it gains a more concrete meaning, it becomes something more fragmented and complex. It is not just one thing, one symbol, but a lot of things […] It acquires a more three-dimensional nature, containing a lot of information: the twisted pillars, the windows blown out, …

As we can observe, the evocative power of the ruins invites the SA to engage in a meaning-making process as the interview develops. Guided first by the abstract meaning traditionally ascribed to the A-Dome, as a symbol of peace, a closer look at the building – this time through the recorded images of the subcam video– offers the SA the opportunity to reflect on the richness of its materiality. This case is an illustrative example of *schematisation* and *pleromatisation*, two complementary processes mediating aesthetic perception. According to Valsiner (2014), schematisation works by reducing the complexity of experience to abstract categories and symbols, thus leading to meaning fixation in the flow of experience. Conversely, “the homogenising role of language symbols is counter-acted by the heterogenising role of [*pleromata*]”, namely “hyper-rich depictions of reality that stand for some other realities” (p. 241). Through pleromatisation new meanings can be created beyond the categorization function of language symbols, something that might be emerging in the post-visit interview. Thus, the detailed description of the A-dome seems to contribute to the emergence of other meanings beyond its official meaning as symbol of peace. More specifically, in focusing on certain aspects of its materiality, the building gains complexity as an intersection point between the idea of peace –officially ascribed to the memorial – and the sense of destruction conveyed through the twisted pillars, the windows blown out, etc.

*4) Afterward: from the weight of the past to the lightness of the future.*

The next day, the FA and the SA went to the Hiroshima National Peace Memorial Hall for the Atomic Bomb Victims (see point “D” in Fig. 1), designed by Kenzo Tange and founded in 2002. We spent some time at the Hall of Remembrance, situated at the lower floor. This circular-shaped hall features a 360-panoramic picture taken right after the bomb from the hypocentre made of 140,000 tiles representing the estimated number of victims who died by the end of 1945. After the visit, carried out without wearing the subcam glasses, the SA wrote down the following impressions as part of his Master thesis (Li, 2022):

“Everything was solemn and peaceful, and my steps were heavy. In addition, the entire site was built underground. In my mind’s eye I realised how deep the history of this place is buried. There was a tension in the air that contrasted with the atmosphere outside. Seeing the names of the destroyed towns inscribed on the walls gave me a heavy feeling. I was stuck in the underground experience for the rest of the day, even when I walked out of the venue. It was at this point that my view of the city changed again, and I felt as if the entire city had also become heavier. It was then that the sound of a song on the radio, praying for peace, jumped into my ears. I felt the city being reborn, which lifted my spirit at the end of the visit […] So this is a city full of hope, not only full of sad memories. That is what the park transmits to me” (p. 41).

This excerpt sums up some of the aspects pervading SA’s experience of the HPMP seen so far. Once more we can see an experience shaped by a constant duality between opposites, between the sad memories of the past and the future’s hope epitomised by the voice of children singing. In this case, we can observe how this experience is manifested through different sensory feelings and embodied metaphors (Lakoff & Johnson, 1980) afforded in turn by the very design and materiality of the site. Thus, the sad memories about the A-bomb victims are associated with “heavy feelings” experienced while being “underground” visiting the “buried history” of the city. Conversely, the SA’s spirit is “lifted” as soon as he reaches the ground level of the park, where he feels the city being reborn, a sensory feeling enhanced by a recorded music that played nearby.^6^ Here we can see an experience similar to the inside/outside transition sensed at the beginning of the visit, although this time taking the opposite direction, going from the inside of the Memorial Hall to a reencounter with the city.

## Discussion: Materiality, Movement, and Meaning

Departing from the possibilities and constraints afforded by memorials’ material and symbolic dimensions, the focus of this single-case study has been to analyse how HPMP is experienced, and more specifically, how different elements of this memorial afford the emergence of affective atmospheres and meaning-making processes as the participant move along and engage with the site. Such a goal could not have been attained exclusively at the level of discourse, through a language-based/mono-modal approach. As Drozdzewski & Birdsall (2019) note, mobility is an enabling methodology in that it makes something apprehensible about memorials that is only possible through movement. In that regard, the use of the subcam, in combination with the post-walk playback interview, has allowed the SA to access his multi-sensory and situated experience, while jointly reflecting with the FA on the footage recorded during his visit. As seen in previous studies (Brescó et al., 2020), this method endows participants with agency when it comes to relate their personal associations, affects and meanings to specific elements of their visit as they watch the visual material recorded at the site.

It is worth noting that, unlike other methodologies used in other studies (Brescó & Wagoner, 2019b; in press), such as go-along interviews, the post-visit interview implies investigating something that took place in the past –even if it is in the immediate past–, thus giving the participant a new opportunity to reinterpret his experience. In this case, the post-visit interview enabled both the researcher and the participant to dig into the meaning-making process associated to different aspects of the memorial as they were shown in the video. As seen, this meaning-making process is to a large degree structured around a string of antinomies which, paraphrasing Moscovici (1993), served as conceptual coat hangers providing socially generated ways of experiencing and interpreting the site. Importantly, these antinomies emerged during the interview from the viewing of the subcam video recorded during the visit, being therefore linked to the physical experience of the memorial and its subsequent visualisation by the SA. More precisely, these antinomies arose when the video showed images that the SA associated with experiences of transition between border zones or experiences of intersection between elements to which he attributed opposing meanings. This occurred at the four points of the visit on which we have focused our analysis.

1. *The memorial entrance*: The presence of the river generated a feeling of crossing a border between two opposed atmospheres (the city’s ordinary life and the HPMP’s area), thus making the initial stretch of the visit (the tree-lined path along the river) to be perceived as a liminal zone between the two. This appears to have called for a meaning-making process whereby the notion of a *sacred* place, applied to the memorial, was set against the idea of *mundane* and everyday life of the city. Being by the river also generated a dichotomy between war, as a man-made artificial thing associated to the memorial, and nature, implicitly associated with peace.
2. *The Cenotaph*: The presence of children contributed to create a happy atmosphere leading the SA to contrast the idea of life, hope and future to the deaths of the past associated with the Cenotaph. The intersection between these dichotomic elements is stressed and objectified through a picture the SA took in front of the monument. This dichotomy is latter recreated through the axial view of the Cenotaph at the foreground (associated with a time near at hand) and the A-Dome (representing a past receding into the background) and expressed through the metaphor of the place as a time machine, thus conveying the sense of intersection between past and present.
3. *The A-Dome*: The dichotomic experience of time shows again when, once close to the A-Dome, the SA highlights the stark contrast between the old ruins and the modern background of the city. This dichotomy between old and new, is complemented by the notion of sacredness attributed to the A-Dome, thus pointing again to an implicit border between the memorial and its mundane surroundings. Finally, the closer look at the A-Dome contributed to the intersection of two contrasting views on the building seen as symbol of peace and as material evidence of war.
4. *The Memorial Hall*: The dichotomy between past/future, death/life emerges again with the SA’s physical transition from the inside of the Memorial Hall –situated beneath the ground– to the park ground level, where the sound of a recorded music contributed to generate an uplifting feeling from the previous sense of heaviness experienced down in the Hall. Similar to the experience at the memorial entrance, there is the sense of crossing a border separating two worlds endowed with completely different meanings, although this time the transition goes from inside to outside the memorial.

As we can see, these dichotomies emerge as a result of the SA’s meaning-making effort at his encounter with different situations during his visit, particularly those leading him to perceive the site in terms of border zones or points of intersection. However, following De Paola et al. (2020), we should be cautious in regarding all antinomies found in our analysis as themata. As Liu (2004) points out, we should differentiate between themata –referred to those “historically embedded presuppositions, culturally shared antinomies, and the deeper logic of social thought” (p. 255)– from those “pragmatic manifestations, or partial reconstructions of the themata in different forms and in the different spheres of everyday life” (p. 256). As elementary dichotomies underlying the social debate around the A-bomb, we can tentatively infer two themata from the string of antinomies found in the post-visit interview.

These two themata correspond to two dimensions –temporal and spatial– involved in the memory politics around the A-Bomb referred to above (Schäfer, 2008; Yoneyama, 1999). The first thema revolves around the problematisation of the past and the future, and more particularly around the tension between a future-oriented recovery and the weight of a painful past in post-war Japan. Cutting through the SA’s experience of the memorial –in front of the cenotaph, the A-Dome, and the Memorial Hall– we can find a sense of temporal duality which leads him to oppose (and, on occasions, combine) the notions death and sadness (associated with the past) and life and hope, which he associates with the future. This temporal duality translates into a spatial dichotomy underlying Hiroshima’s urban renewal, giving rise to a second themata referred to the tension between the old and the new. This dichotomy –expressed at the beginning of the visit and in front of the A-Dome– is especially linked to the experience of border zones separating two different areas (inside vs. outside the memorial) to which the SA associates with a sacred and a mundane world, respectively.

Combined together these two themata help us to reflect on the problematisation of time and space in the city of Hiroshima. However, one might ask to what degree the existence of different urban topographies linked to dissonant temporalities, as Yoneyama (1999) puts it, helps us to further reflect on the A-bomb or, on the contrary, contributes to limiting the memories and the debate through its spatial and temporal containment within the memorial’s boundaries. As Yoneyama (1999) warns us, “the containment of memories of destruction obscures other contemporary realities: namely, that the nuclear horror may in fact be present everywhere outside this museumized site, that the world may be thoroughly contaminated by nuclear weapons” (p. 52).

## Conclusions: Beyond the Hiroshima Peace Memorial Park 

Through this article we aimed to address Tange’s question about what crosses people’s minds when visiting the HPMP. To be sure, a defining aspect of HPMP is its location, close to the explosion’s hypocentre. When a memorial is constructed at the site of the traumatic events it is already affectively charged with the site’s history^7^ (see Wagoner & Brescó 2022). Despite addressing this question through a single-case study involving three non-Japanese researchers (one of them as a participating subject), we can conclude by highlighting the enormous power that a place like the HPMP exerts on those who visit it, regardless of their background. As the SA commented at one point during the visit, when sharing his feeling that the memorial was speaking to him through the voices of the victims, “even though I am Chinese –and, you know, Japanese troops attacked China–, I feel that these people [the victims] could also be my grandma or grandpa telling me something”.

As a paradigmatic example of what Bull & Hansen (2016) call a *cosmopolitan mode of remembering*, the HPMP is inextricably linked to a transnational memory discourse anchored in a set of iconic artefacts –such as the A-Dome – for remembering the past vis-à-vis potential futures we would like to build or avoid as human beings. The global dissemination of images featuring the mushroom cloud, devastated cities, the nuclear reactor, or the radiation symbol have forever changed the way humanity imagines (and questions) its future. As Jasanoff & Kim (2009) examine through the notion of *sociotechnical imaginaries*, imagination of possible futures –whether desirable horizons worth attaining or grim scenarios to be avoided– is becoming increasingly shaped by scientific and technological advances, such as the discovery of nuclear energy.

Yet, imagination about the future is also largely dependent on how we remember the past, and vice versa (Brescó, 2017; Brescó & van Alphen, 2021). Thus, as Yoneyama (1999) rightly notes, the preservation of the A-Dome ruins, as an eternal reminder forever inscribed in the memory of humanity, “can be trusted only if one believes that the present state of things will remain in equilibrium” (p. 52). In that respect, she asks, “Are visitors to the site prompted to wonder about the possibility of future similar destructions?” (p. 52). While these ruins stand as iconic historic evidence of the atomic destruction, Yoneyama (1999) warns us that the musealisation and sacralisation of the A-Dome’s ruins also contributes to bringing this building “into an ahistorical and almost naturalized past […] derailed from the secular course of history” (p. 51). At the same time, this author goes on to say, “the Dome’s stark contrast to its background scenery, a magnificently recovered urban space, assures people of today’s peaceful, prosperous, and clean world” (p. 52). Agreeing with the author, we think that the remembrance of the horror caused by the A-bomb should be present beyond the memorial’s spatial and temporal boundaries, in the knowledge that the danger of nuclear destruction is still looming all over the world.

## Data Availability

The datasets generated during and/or analysed during the current study are available from the corresponding author on reasonable request.

## Footnotes

1 The second atomic bomb, dropped three days after on Nagasaki, would lead to the surrender of Imperial Japan on August 15, formally signed on September 2 abord the USS Missouri.

2 The first author is in debt with Prof. Minami’s valuable advice on the fieldwork at HPMP, and with Prof. Jaan Valsiner for making Prof. Minami’s acquaintance possible.

3 While the SA was introduced to the subcam methodology prior to the fieldwork, he was blind to most of the theoretical concepts that have informed the subsequent analysis of the data. This is particularly true for the notion of *themata* insofar as the use of this concept by the first and third authors emerges bottom-up only later when analysing the interview. This is evidenced in that the SA’s analysis of his own experience at HPMP in his Master thesis (see Li 2022) takes on an autoethnographic approach and is guided by other theoretical concepts related to the notion of Genius Loci. The inclusion of his experience at HPMP in his Master thesis was a decision made by the second author after the fieldwork with the permission of the first author.

4 The HPMP is located in the northern part of one of the several islets formed by the Ota’s River six channels emptying into the Seto Inland Sea.

5 Taken as the aiming point for the A-bomb due to its easily recognisable T-shaped structure, the bridge manged to stand after the explosion and, following some repair works, it remained in service until being replaced by a new replica in 1983.

6 Installed in 2001 in front of one of the trees transplanted in within the park in 1973, the audio system plays the song “Aogiri no Uta” (Chinese Parasol Tree) sung by a nine-year old girl.

7 Some authors (Norberg-Schulz, 1979; Suzuki, 2017) study this affectively charged atmosphere through the ancient Roman notion of *Genius loci*, namely the spirit of a place, see Li (2022) for a recent revision of this concept.
